# Tissue effects of advanced energy devices: a systematic review and descriptive analysis of vascular sealing and lateral thermal damage in human tissue

**DOI:** 10.1007/s00464-026-13485-y

**Published:** 2026-10-13

**Authors:** D. Marshall, N. Kahar, C. Challand, L. Wyld

**Affiliations:** 1 University of Sheffield, Western Bank, Sheffield, S10 2TN UK; 2 Sheffield Teaching Hospitals NHS Foundation Trust, Sheffield, UK

**Keywords:** Advanced energy, Surgical devices, Electrosurgery, Vessel sealing, Thermal spread

## Abstract

**Introduction:**

Advanced energy devices such as bipolar and ultrasonic devices are used ubiquitously in all aspects of surgery. Despite studies demonstrating differences between animal and human tissues, existing human studies have only been conducted in terms of operative outcomes such as blood loss, with a paucity of data on vessel sealing and lateral thermal spread. This study aims to consolidate all existing literature on these characteristics for each device in human tissues to inform clinician decision making.

**Methods:**

A systematic review of the literature was carried out using PRISMA guidelines. Papers which discussed vessel sealing or lateral thermal spread with any methodology were included. Exclusion criteria included animal studies, conference abstracts or papers where full text English language papers were not available. Risk of bias was assessed using ROB2, ROBINS-I v2, CASP case–control and JBI case series checklists as appropriate for the study design. A descriptive analysis was undertaken.

**Results and discussion:**

Across the twenty-eight included studies, most vessel seals with bipolar and ultrasonic devices exceeded physiological burst pressures. Seal failures occurred unpredictably. Lateral thermal spread varied widely (0–7.64 mm) depending on the device and tissue examined as well as the testing methodology and definitions of lateral thermal spread. Due to substantial variation in study methods, direct statistical comparisons were not possible and a reliable device-to-device comparison cannot currently be made from the available literature. This highlights the need for standardized testing methodology.

PROSPERO registration and full protocol availability: CRD420251239034

**Supplementary Information:**

The online version contains supplementary material available at https://doi.org/10.1007/s00464-026-13485-y.

## Introduction

Advanced Energy devices are used ubiquitously in all aspects of surgery. Advanced bipolar devices (ABP) and ultrasonically activated devices (USAD) are the most commonly used. Substantial literature exists relating to the operative outcomes in specific procedures comparing traditional electrocautery with the use of such advanced energy devices, with clear advantages in intra-operative blood loss and operative time for many procedures and specialties [1]. Despite these benefits, it is widely acknowledged that the main complications associated with these devices is collateral thermal injury to adjacent tissues either through direct thermal spread, electrical coupling or from residual heat in the device when touched to other tissues after activation [2, 3]. The incidence of collateral injury was reported as 3553 injuries and 178 deaths in the US between 1994 and 2014 [4]. This has prompted institutions such as Society of American Gastrointestinal and Endoscopic Surgeons (SAGES) to generate programmes designed to increase education about electrosurgery [4].

Choice of devices between surgeons is often thought to be based on personal or institutional experiences rather than deep understanding of the characteristics of each device. A study by Braungart and colleagues showed that clinician understanding of such devices is variable, with 15% of 184 consultant surgeons surveyed unaware that the Harmonic scalpel remains at > 60 °C for up to 45s after use [5].

There is a paucity of evidence relating to lateral thermal spread and vessel sealing in human tissues with most studies being carried out on animal tissues with a focus on bursting pressure in vessels correlated with size of these vessels. Several studies have shown great variation between bursting pressures of animal tissues and human tissues. Notably, porcine renal arteries having a significantly higher bursting pressure than any human cadaveric vessel in one particular study [6], with another suggesting correlation between bursting pressure and collagen:elastin ratio in the vessels which are being sealed [6, 7]. Additionally, there have been case reports of vessel sealing failure with such devices in patients with collagen disorders [8].

Through systematic review of the literature, this study will collate all existing data on lateral thermal spread and vessel sealing in human tissues and aims to identify these characteristics for each device.

## Methods

A systematic review of the literature was carried out in accordance with the Preferred Reporting Items for Systematic Reviews and Meta-Analyses (PRISMA) guidelines after registration on PROSPERO. Ovid Medline, Embase and Scopus were systematically searched using the terms “bipolar device”, “ultrasonic scalpel”, “lateral thermal spread” and “burst pressure” from inception to October 2025. Full search methods are available in Online Appendix 1 and 2. Abstracts were screened independently by 2 reviewers with arbitration of any conflicts by a third reviewer. Inclusion criteria included any paper discussing analysis of lateral thermal spread (any methodology) or bursting pressures of any blood vessels in human tissues. Exclusion criteria included any paper discussing only clinical outcomes (such as mean blood loss or operative time), animal studies or where the paper was a case report, presented abstract or letters. Bibliographies of studies including in full-text screening were also screened to identify any further papers however no further papers of relevance were identified. Exclusion criteria included animal studies, lack of relevant datasets, studies which were conference abstracts only or where full-text English language papers were not available.

One reviewer extracted data from each study using pre-defined criteria and included any data regarding lateral thermal spread or blood vessel bursting pressure. Data extracted included basic study information including design, participant numbers and outcome measures. Data regarding the devices used in each study including both broad category and specific device (where specified) were collated. For papers concerning vessel sealing the number of participants as well as sample size were extracted. Methodology, type of vessel, fixed and unfixed vessel sizes as well as bursting pressure were collated. For papers concerning lateral thermal spread the methodology of assessment, definitions of thermal injury and reported values for lateral thermal injury were collated alongside device and study characteristics. There was significant heterogeneity in reporting and therefore a meta-analysis was not possible. Synthesis and descriptive analysis of data was pooled to tissue and device type.

### Risk of bias assessment

A risk of bias assessment was carried out for all studies by one reviewer. Randomized controlled trials were assessed using the ROB2 too l [9], non-randomized trails the ROBINS-I v2 [10] tool and case series with the Joanna Briggs institute (JBI)checklist for case series [11]. All remaining studies including case–control, retrospective and cadaveric studies were assessed using the Critical appraisal skills programme (CASP) checklist for case–control studies [12]. For retrospective and cadaveric studies, the CASP checklist was utilised to give a generic framework to assessing the different aspects of potential bias. An overall judgement on risk of bias was made for each article assessed with the JBI and CASP checklists as this is not a formal output from these tools.

## Results and discussion

**Fig. 1:**
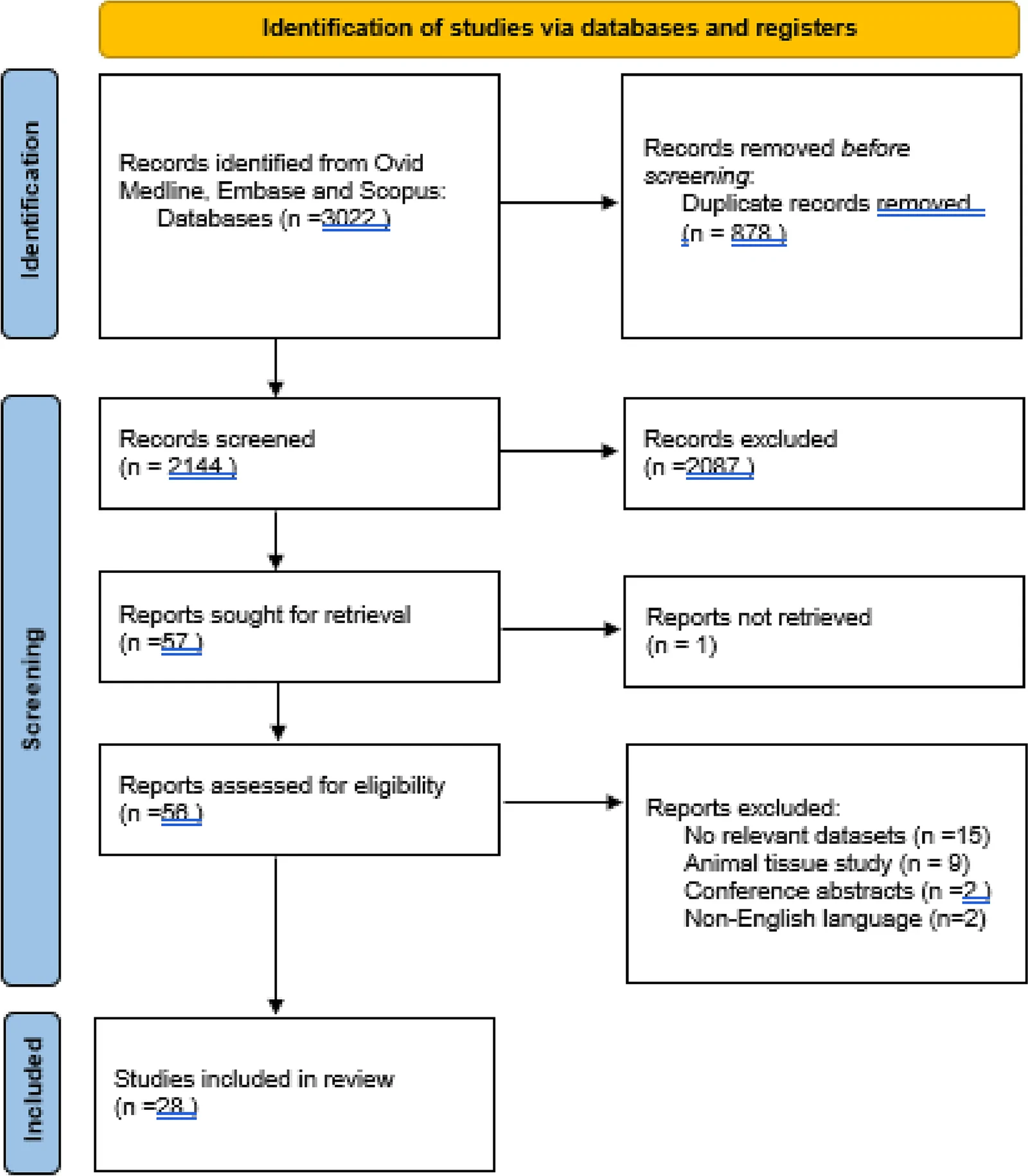
Preferred reporting items for systematic reviews and meta-analyses (PRISMA) flowchart

In total 2144 abstracts were screened after deduplication. Of these 57 were included for full-text screening and of these, 28 papers were eligible for inclusion (Fig. 1). Of these papers, 12 included datasets relating to bursting pressure and 20 contained information of lateral thermal spread. Four of these papers contained both lateral thermal spread and bursting pressure data. Risk of bias assessments are described in Online Appendix 3 with the majority of papers showing low risk of bias (*n* = 17), moderate risk/some concerns (*n* = 5), high/serious risk (*n* = 5) and 1 paper at critical risk of bias. The papers at high and critical risk of bias were assessed as so due to failure to account for confounding variables or methodological concerns with the randomization process.

### Bursting pressure

Of these 28 papers, 12 papers contained datasets relating to bursting pressure of vessels. The descriptive statistics of these studies are displayed in Online Appendix 4. Overall, these papers represent 320 patients and 939 individual bursting pressure samples with the majority of studies being single centre RCTs or cohort studies.

**Fig. 2:**
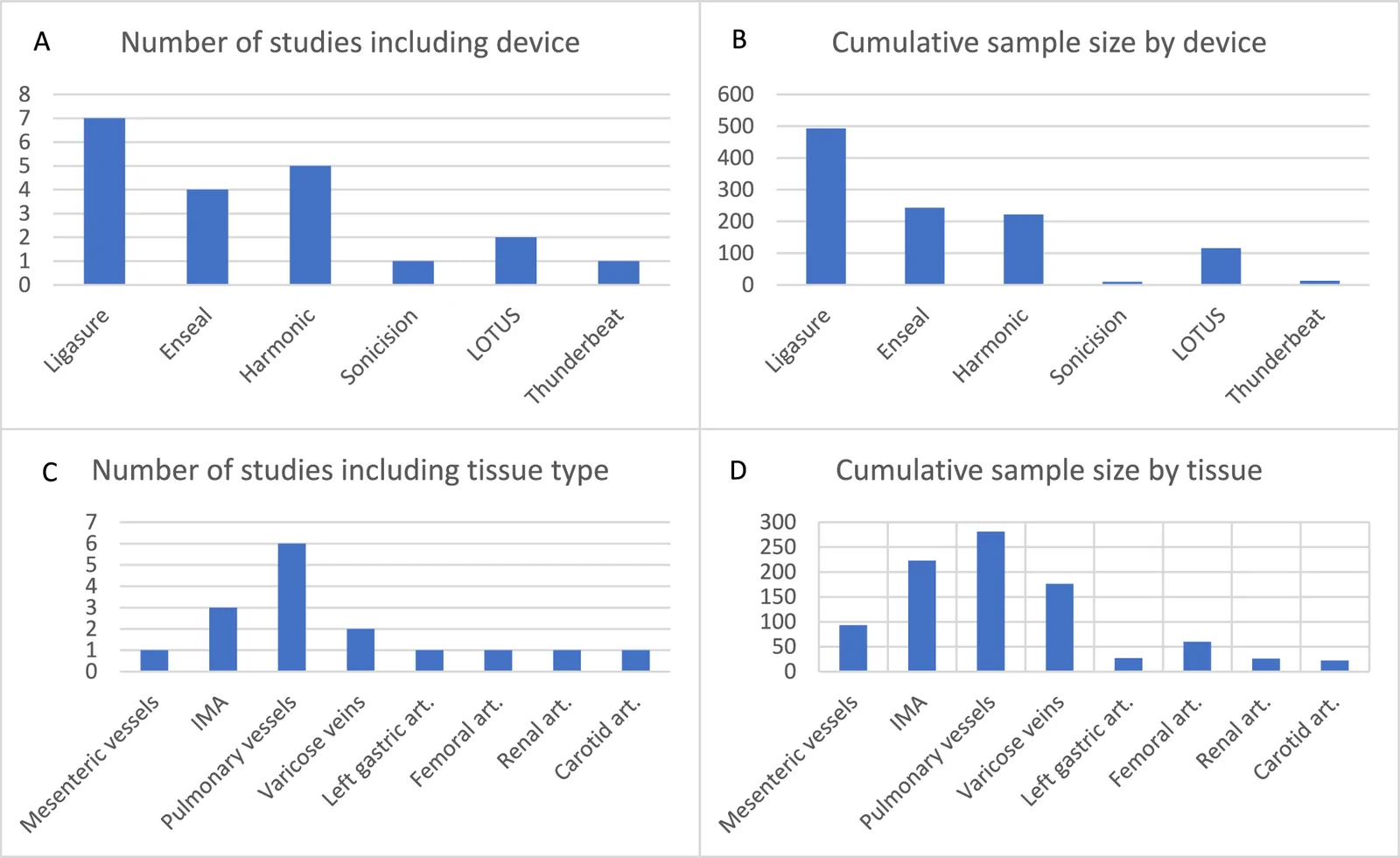
Simple bar charts demonstrating: **A** the number of studies which included each device in testing, **B** corresponding cumulative sample sizes for these studies, **C** the number of studies including different tissue types, **D** corresponding cumulative sample sizes for these studies. *IMA* inferior mesenteric artery devices (Manufacturer): Ligasure (Medtronic), ENSEAL (Ethicon), Harmonic (Ethicon), Sonicision (Medtronic), LOTUS (BOWA medical), THUNDERBEAT (Olympus)

The most commonly investigated type of device was the advanced bipolar Ligasure (Maryland, dolphin tip and Atlas) (Medtronic), which were included in the analysis of 7 papers and represented a cumulative sample size across all studies of 493. The most common tissue type was pulmonary vessels, which were included in 6 studies and had a cumulative sample size of 281 as shown in Fig. 2.

Many of these studies are single centre studies with small numbers of patients with variable study methodology and tissue types. Of note, there is only a single paper assessing Sonicision (Medtronic) and THUNDERBEAT (Olympus) with regard to bursting pressures in human tissues.

Table 1 shows a summary for each of the included studies and demonstrates the heterogeneity in experimental design, particularly with regard to tissue, vessel size and outcome reporting.

**Table 1:** Summary of each included paper

Authors	Tissue	Summary of findings
Kajiyama et al. (2005) [13]	Left gastric artery	Vessel diameter not reported. Mean bursting pressures 214 mmHg (SD 13.1) range 51–300 mmHg. Maximum pressure of 300 mmHg for transducer used
Ching et al. (2008) [14]	Varicose veins	Compared LOTUS and Harmonic scalpel. Split vessels into 1mm increments from 2 to 6 mm. Reported Busting pressures as Median (IQR). Overall Median BP for LOTUS 149 mmHg and 109 mmHg for harmonic. Decreasing bursting pressure for increasing vein diameter
Tsunezuka et al. (2010) [15]	Pulmonary vessels	Grouped vessels by < 5 mm (small) or > 5mm (large). With Ligasure small arteries burst pressure 607 mmHg (SD 78), large 447 mmHg (SD 119). Significance between vessel sizes
Noble et al. (2011) [16]	Mesenteric vessels	Unnamed mesenteric vessels with mean diameter 1.06 mm (SD 0.70). Compared LOTUS, Harmonic ACE and Ligasure with mean burst pressures of 1170 mmHg (SD 440), 1470 mmHg (SD 670), 1510 mmHg (SD 740) respectively. No significant difference between instruments
Lesser et al. (2013) [17]	Pulmonary vessels	Grouped vessels into 3–5 and 6–8 mm sizes with mean bursting pressures of 315 mmHg (SD 213.1) and 156 mmHg (SD 42.5) respectively. Note incomplete fusion of sealed vessels showing intima and media invaginated
Latimer et al. (2014) [6]	Multiple sites	Vessel diameters not reported. Multiple different named vessels sealed with Ligasure in cadaveric specimens with mean burst pressures as below: Deep femoral—529 mmHg (SD 246) Femoral artery—428 mmHg (SD 206) Carotid artery—423 mmHg (SD 131) Iliac artery—459 mmHg (SD 251) IMA—564 mmHg (SD 271) Renal artery—480 mmHg (SD 226) Splenic artery—170 mmHg (SD 50) Pulmonary artery—320 mmHg (SD 173) Pulmonary vein—353 mmHg (SD 183)
Liberman et al. (2014) [18]	Pulmonary vessels	Pilot study for 2015 paper, below. Compared harmonic ACE, EnSEAL Trio, Ligasure and THUNDERBEAT on vessels with a mean diameter of 7.44 mm with mean bursting pressures of 415 mmHg (SD 361.18), 133.67 (SD 105.89), 214mmHg (SD 170.21) and 847.96 (SD 682.52) respectively
Toishi et al. (2014) [19]	Pulmonary vessels	Compared EnSEAL Trio, Ligasure and Harmonic ACE on vessels with a mean diameter of between 4.3 and 4.6 mm with mean bursting pressures of 309 mmHg (SD 177), 351 mmHg (SD 249), 259 mmHg (SD 129) respectively. Also split vessels into < 5 mm or > 5 mm with significantly lower burst pressures in the larger vessels overall but not presented between devices
Bibi et al. (2014) [20]	IMA	Assessed EnSEAL on vessels with a mean diameter of 4 mm sealed both in and ex vivo. Found mean bursting pressure of 862 mmHg (SD 282) with no significant difference between in and ex vivo seals
Liberman et al. (2015) [21]	Pulmonary vessels	Continuation of 2014 pilot study, above. Assessed Harmonic ACE+ on vessels with a mean diameter of 5.2 mm finding a mean bursting pressure of 333 mmHg (SD 231.4)
Balachandran et al. (2017) [22]	IMA	Assessed the use of an angled EnSEAL instrument (G2) on vessels with a mean diameter ranging from 3.48 to 3.69 mm between groups (0-, 30-, or 60-degrees instrument angulation). Identifying mean bursting pressures of 1604 mmHg, 1507 mmHg and 1478 mmHg, respectively with no significance between groups
Miralles et al. (2023) [23]	Varicose veins	Compared Ligasure with Sonicision on vessels with a mean diameter ranging from 2.85 to 3.2 mm between groups and identifying bursting pressures of 801.5 mmHg (SD 234.88) and 637 mmHg (SD 320.61) respectively

*SD* standard deviation

There was significant heterogeneity in the devices, power setting, tissues and testing methods employed by all studies, precluding meta-analysis or direct comparison of data.

There is a wide variation in reported bursting pressures both within individual studies and between studies for different devices and tissue type. Liberman and colleagues [18] demonstrated a range of vessel bursting pressures of between 0 and 579.6mmHg for vessels sealed with the Ligasure (Medtronic) Dolphin tip instrument. Comparatively, Noble and colleagues reported mean bursting pressures of unnamed mesenteric vessels sealed with the Ligasure Atlas device (Medtronic) as 1510 mmHg. The wide variation in vessel diameters and range of bursting pressures for each device is summarised in Table 2. For studies which have reported mean and standard deviation, but no range. A range has been extrapolated from the mean plus two standard deviations, assuming a normal distribution to facilitate comparison. The (arbitrary) values for which these devices are marketed is often three times physiological systolic blood pressure, however this is not necessary for the sealing of all vessels, as described by any study looking at varicose saphenous veins or pulmonary vessels for example.

**Table 2:** Ranges of vessel diameter and bursting pressures per device type

	Vessel diameter range (tissue)		Bursting pressure range (tissue)	
	Lowest value (mm)	Highest value (mm)	Lowest value (mmHg)	Highest value (mmHg)
Ligasure	1.81 (pulmonary vessels) [18]	14.52 (pulmonary vessels) [18]	60 (pulmonary vessels) [18]	1510 (mesenteric vessels) [16]
ENSEAL	1.5 (IMA) [24]	12.89 (pulmonary vessels) [18]	53.13 (pulmonary vessels) [18]	2390 (IMA) [24]
Harmonic	1.7 (pulmonary vessels) [21]	12.71 (pulmonary vessels) [18]	84 (pulmonary vessels) [21]	1415 (pulmonary vessels) [21]
LOTUS	2–3 (varicose veins) [14]	5–6 (varicose veins) [14]	61* (varicose veins) [14]	2050** (mesenteric vessels) [16]
THUNDERBEAT	4.46 (pulmonary vessels) [18]	10.15 (pulmonary vessels) [18]	237.08 (pulmonary vessels) [18]	2871.31 (pulmonary vessels) [18]

Where vessel sealing failed (i.e. burst pressure of 0mmHg—these have been excluded)

For studies which have reported mean and standard deviation, but no range. A range has been extrapolated from the mean plus two standard deviations, assuming a normal distribution, to facilitate comparison. *Lower quartile only reported **extrapolated from mean value +2× SD

There are numerous papers which document bursting pressure for animal tissues, with porcine vessels including renal and carotid most commonly utilised for this indication [25–29]. To the authors knowledge there is only one paper which directly compares vessel sealing and bursting pressures in porcine and human tissues. Latimer and colleagues found that porcine renal arteries have significantly higher bursting pressures than any human cadaveric vessels and that carotid or renal arteries serve as the best model for human tissues [6].

The mean and median bursting pressures reported in the human vessel studies are congruent with animal tissues in that the majority of studies show mean and median bursting pressures for all devices significantly above physiological blood pressure levels. This also correlates with studies focusing on clinical outcomes in which operative blood loss is lower with energy devices than with traditional haemostatic methods [1] or studies in which named vessels are divided with energy devices only, as in the study by Introini and colleagues in the closure of in vivo renal arteries using ENSEAL (Ethicon) alone with no major haemorrhagic complications noted [30].

### Vessel sealing failure

Both ABPs and USADs are associated with seal failures. Ching and colleagues demonstrated median burst pressure values below physiological levels in varicose vein seals using both the LOTUS (BOWA Medical) and Harmonic scalpel (Ethicon) [14]. It is also notable that many studies report a range of pressures which include bursting pressures below physiological levels or demonstrating complete seal failures [13, 14, 17, 18, 21]. Despite being a small proportion of vessel seals, and vessel with a seal bursting pressure below physiological levels or complete seal failures are clinically important as they could lead to catastrophic haemorrhage as noted by Martin et al. who had an effective seal rate of 99.8% in colorectal major vessel resections, however 2 patients required conversion to laparotomy for control of major haemorrhage [31]. The study by Liberman et al. tested vessels of with a range of 1.81–14.52 mm. The vessels which had seal failures measured 6.78 mm, which is below the marketed indication of 7 mm. There is no comment on whether this vessel contained atherosclerotic disease [18] which is a noted contraindication for the use of bipolar devices and leads to seal failures as demonstrated by the study by Bibi et al. with sealing failure of an IMA with atherosclerotic disease [20]. There is no contraindication to the use of USAD in atherosclerotic vessels however there are no reports on this technology’s efficacy in such vessels either.

There is conflicting data on whether burst pressure is associated with vessel diameter with some studies supporting this [15, 17, 19] and some studies finding no correlation [20, 23]. This is likely due to the complexity of different tissue and vessel types and does not demonstrate clear association with vessel diameter and device type.

Latimer et al. directly compared cadaveric seals to that of porcine tissues in an effort to correlate burst pressure with collagen:elastin ratio of tissues. They found that collagen:elastin ratio had a larger influence on vessel bursting pressure in vessels <5mm in size, but less of a pronounced effect in larger vessels. This correlated with significantly differing bursting pressure values between vessels around the human body, with splenic arteries showing a mean burst pressure of 170 mmHg (50 SD) and inferior mesenteric arteries a mean burst pressure of 564 mmHg (271 SD). This may partially explain the pathophysiology describing haemostatic failure of the Ligasure device (Medtronic) in a case report by Jalink et al. a patient with vascular Ehlers-Danlos [8].

The mechanism of bursting may be due to incomplete seals or invagination of components of the vessel wall. In studies by Liberman et al. 33% of pulmonary vessels sealed with the Harmonic ACE (Ethicon) features invagination of the media and intima, with only 66% of vessels showing complete seals of all layers of the vessel [21] with Lesser et al. demonstrating adventitial fusion only in all pulmonary arteries sealed with the Ligasure device (Medtronic) [17].

It is not clear which device gives the most reliable seal when considering tissue type or vessel size due to underpowered studies and heterogeneity There is clear contraindication to the use of bipolar devices in atherosclerotic vessels, and perhaps evidence to suggest contraindication in collagen disorders, however there is no evidence either way for the use of USADs in such tissues.

### Lateral thermal spread

Twenty papers were identified with data on any method of lateral thermal spread analysis, including a total of 865 patients and 1761 samples in the energy device arm(s) of the study. The descriptive statistics of these studies are shown in Online Appendix 5.

Table 3 highlights the differences between methodologies for testing the degree of lateral thermal spread. The most common technique was histological Haematoxylin & Eosin staining (H&E); however, the method of interpretation was heterogenous, with some studies stating that the edge of the thermal injury was clearly visualised and others documenting clear cellular architectural or collagenous changes. Collagenous changes are visualised more clearly with Elastica Van Gieson (EVG) and Masson’s trichrome stains which were utilised in 4 studies though the differences in lateral thermal injury measured with H&E vs collagenous stains were not commented upon in any study.

**Table 3:** Study methodologies and definitions of lateral thermal spread

Authors and year	Method	Definition(s)
Gorginzadeh et al. (2024) [32]	H&E	Altered staining in the fibromuscular tissue
Mihanovic et al. (2021) [33]	H&E	To the edge of unchanged tissue
Turkan et al. (2019) [34]	H&E	Loss of collagen fibre structure
Yoshiya et al. (2018) [35]	Masson’s Trichrome	Farthest point stained by red by Masson's
Choi et al. (2018) [36]	H&E	To the edge of unchanged tissue
Caltekin et al. (2017) [37]	Masson’s Trichrome	To the edge of damaged or necrotic tissue
Balachandran et al. (2017) [22]	Not defined	Not defined
Pogorelic et al. (2017) [38]	H&E	To edge of unchanged tissue
Adamczewski et al. (2015) [39]	H&E and thermography	To the edge of unchanged tissue, 42C isotherm
Bibi et al. (2014) [20]	Not defined	Not defined
Mannelli et al. (2014) [40]	H&E	Complex system rating cellular changes and fibrocollagenous changes into zones of injury
Zhu et al. (2012) [41]	H&E	Cellular architectural changes
Druzijanic et al. (2012) [42]	H&E	To the edge of unchanged tissue
Hanby et al. (2011) [43]	H&E	Not defined
Noble et al. (2011) [16]	H&E, EVG	Not defined
Brzezinski et al. (2011) [44]	Thermography	42 °C isotherm
Zorn et al. (2010) [45]	H&E and TUNEL	Not defined
Modi et al. (2009) [46]	H&E	Cellular architectural changes and homogenization of connective tissue
Ching et al. (2008) [14]	H&E, EVG	Cellular architectural changes and elastic fibre morphology
Kawamata et al. (2001) [47]	Thermography	43 °C isotherm

TUNEL (Terminal deoxynucleotidyl transferase dUTP nick end labelling) staining highlights apoptotic cells through detection of DNA strand breaks. Notably and as shown in Table 4 in the study by Zorn et al. [45] there was a difference in the values quoted for lateral thermal injury for H&E and TUNEL staining, with TUNEL showing a slightly greater degree of injury than H&E but no analysis of significance was reported.

**Table 4:** Summary of study including main outcome findings

Authors and year	Summary of study and findings
Gorginzadeh et al. (2024) [32]	Checked margins anterior, posterior, left and right lateral colpotomy with Harmonic. Means ranged from 2.92 to 3.23 mm
Mihanovic et al. (2021) [33]	Primarily assessed new vs. old harmonic scalpels during appendicectomy. Median lateral thermal damage on appendix and mesoappendix ranged from 0.1 to 0.2 mm with no significance between new and old or appendix base vs mesoappendix
Turkan et al. (2019) [34]	Compared mono/bipolar to Ligasure in breast lumpectomy. No significant differences found. Median lateral spread for Ligasure 1 mm
Yoshiya et al. (2018) [35]	Used a variety of stains to delineate elastic, collagen and smooth muscle fibre changes in Ligasure sealed pulmonary vessels. Median values ranged 0.187 and 0.236 mm for small and large arteries and veins without significance between groups
Choi et al. (2018) [36]	Compared monopolar to an ultrasonic hook during colpotomy. Median USAD lateral thermal spread 0.95 mm, this was significantly less than monopolar
Caltekin et al. (2017) [37]	Compared Ligasure and EnSEAL on proximal and distal fimbrial tubes. Mean lateral thermal spread measurements between devices on distal and proximal tubes were 2.128 mm and 2.294 mm for Ligasure and 1.671 mm and 1860 mm respectively with no significance found between groups
Balachandran et al. (2017) [22]	Compared articulating EnSEAL device at different angulations when sealing IMAs. Mean lateral thermal spread ranged from 0.64 mm to 0.89 mm with no significant differences between groups
Pogorelic et al. (2017) [38]	Compared the Harmonic, Ligasure and MiSeal devices on appendix base and mesoappendix specimens. Median lateral thermal spread for Harmonic was 0.08 and 0.1 mm, Ligasure 0.13 mm and 0.16 mm, MiSeal 0.08 mm and 0.1 mm for mesoappendix and base of appendix, respectively. Only significant finding was increased lateral thermal damage for Ligasure on both tissues compared to other devices
Adamczewski et al. (2015) [39]	Compared Harmonic, Sonosurg, Ligasure and Thermostapler at different power settings. Correlated thermography to histological changes. Found No significant difference in the histological width of thermal injury or diameter of 42C isotherm between groups. Mean histological range of injury was 0.26–0.36 mm. Mean diameter of 42C isotherm was 9.62–12.8 mm
Bibi et al. (2014) [20]	Assessed lateral thermal damage on IMAs sealed in vivo with EnSEAL. Mean lateral thermal spread was 0.57 mm and was not associated with vessel size
Mannelli et al. (2014) [40]	Assessed thermal changes in excised laryngeal mucosa using monopolar vs. harmonic vs. CO_2_ laser and impacts on surgical margins. Found mean lateral thermal spread for harmonic of 0.862 mm, this was significantly less than monopolar but more than CO_2_ laser
Zhu et al. (2012) [41]	Compared mono/bipolar to Ligasure and BiClamp in myometrial tissue. Ligasure had widest mean area of lateral thermal spread (5.08 mm) significance reached against BiClamp (3.83 mm) but not significant against mono/bipolar
Druzijanic et al. (2012) [42]	Compared monopolar, Ligasure and harmonic in vivo peritoneum. Found the mean lateral thermal damage of monopolar, Harmonic scalpel (output power 3), Harmonic scalpel (output power 5) and Ligasure were 0.215mm, 0.090 mm, 0.127 mm and 0.144 mm, respectively. Significance between all results other than between harmonic at max power and Ligasure
Hanby et al. (2011) [43]	Compared harmonic vs. CO2 laser in cadaveric tongue resection. Found mean harmonic thermal damage of 0.69 mm, significantly more than CO2 laser
Noble et al. (2011) [16]	Compared LOTUS, harmonic and Ligasure on unnamed mesenteric vessels. Mean thermal damage 3.37 mm for Ligasure, 2.18 mm for LOTUS and 1.95 mm for Harmonic. Significance reached for Ligasure vs either USAD
Brzezinski et al. (2011) [44]	Assessed the use of Thermostapler with thermography during thyroidectomy. Found maximum width of 42°c isotherm for Thermostapler was 3.79 mm at 30W and 4.9 mm at 50W. No significant difference from monopolar or bipolar at the same energy levels, however significantly more between energy levels
Zorn et al. (2010) [45]	Primarily assessed effects of cold saline irrigation when using EnSEAL on lateral thermal injury. H&E showed mean lateral injury of 0.31mm and 0.98 mm for EnSEAL plus irrigation and EnSEAL alone, respectively. TUNEL staining showed lateral injury of 0.39 mm and 1.12 mm for these same groups. Significance reached with cold irrigation vs. no irrigation
Modi et al. (2009) [46]	Compared monopolar, harmonic and coblation device in tonsillectomy. Found mean thermal injury of 0.68 mm for harmonic. No significant difference between groups
Ching et al. (2008) [14]	Compared torsional and linear USADs in varicose veins. Found mean lateral thermal spread of 1.479 mm for torsional shears and 1.116 mm for linear with significance found between groups
Kawamata et al. (2001) [47]	Thermography using harmonic showed a 6mm 43 °C isotherm during brain resection. This was less than bi/monopolar (10 and 15 mm, respectively)

When considering methods of assessing lateral thermal spread at a cellular level, the areas of tissue subjected to coagulative necrosis show clear architectural disruption and should be easily identified on simple H&E staining. There is however a peripheral zone in which cells may undergo either cellular stasis or delayed apoptosis either due to direct heat related injury or molecular stress signalling [48]. EVG, Masson’s trichrome and Picosirius red staining (amongst others) detail collagenous thermal changes as opposed to the architectural changes seen with H&E, thereby capturing cells which may undergo later apoptosis. Direct DNA damage methodologies such as TUNEL staining also attempt to capture this tissue which is non-viable but not obviously necrosed with simple H&E staining. DNA staining techniques are highly specialised, expensive and time-consuming processes. Contrastingly simple collagen staining methods such as Masson’s trichrome or Picrosirius red staining are significantly cheaper and simpler to process.

Three studies utilised thermography. Two studies used a 42 °C isotherm and 1 study a 43 °C isotherm. Notably the thermographic data suggested a much wider (histologically the mean range of injury was 0.26–0.36 mm. Mean diameter of 42 °C isotherm was 9.62–12.8mm) area of tissue temperature at the temperature which is associated with thermal injury than correlated with histological changes with H&E staining [39]. The study by Brzezinski and colleagues also demonstrated thermography when utilising the Thermostapler (EMED) in thyroidectomy with widths of 3.79 mm and 4.9 mm at 30W and 50W power respectively but did not correlate with histological findings [44]. There is clearly a disparity between simple H&E staining and TUNEL or thermographic data in the assessment of lateral thermal spread. This may be due to steam formation which does not directly correlate with tissue injury unless the steam heats that adjacent tissue.

There is significant variation in the ranges of measurements for lateral thermal spread both within studies and between studies. As seen in Table 5, one study demonstrated lateral thermal spread of a Ligasure device (Medtronic) of 0.04 mm with another study measuring lateral thermal spread of 7.64 mm. For studies which have reported mean and standard deviation, but no range. A range has been extrapolated from the mean plus two standard deviations to facilitate comparison. It is difficult to directly compare these data due to the heterogeneity in methodology assessing lateral thermal spread, device and tissue types utilised. Although statistical testing is not possible due to heterogeneity of data, there is no clear correlation between USAD or ABP with lateral thermal spread, with similar ranges seen between both technologies. Notably, several studies have quoted lateral thermal spread in excess of 1 mm for commonly utilised devices such as the harmonic, which aims to minimise lateral thermal spread.

**Table 5:** Range of histological values of lateral thermal spread by device

Device	Lowest value/mm (method) [tissue]	Highest value/mm (method) [tissue]
Harmonic (Ethicon)	0.02 (H&E) [mesoappendix] [38]	5.79* (H&E) [colpotomy] [32]
Ligasure (Medtronic)	0.04 (H&E) [Mesoappendix] [38]	7.64* (H&E) [myometrium] [41]
Sonosurg (Olympus)	0.23* (H&E) [thyroidectomy] [39]	0.42*(H&E) [thyroidectomy] [39]
Thermostapler (EMED)	0.22* (H&E) [thyroidectomy] [39]	0.41 (H&E) [thyroidectomy] [39]
ENSEAL (Ethicon)	0 (Not defined) [IMA] [20]	3.369 (H&E) [fimbral tubes] [37]
LOTUS (BOWA Medical)	0.11* (H&E, EVG) [mesenteric vessels] [16]	3.79* (H&E, EVG) [mesenteric vessels] [16]

Definite data points were not available for all devices and so when a reported standard deviation and mean are given, maximum values have been extrapolated from this data assuming a normal distribution of data

*Value extrapolated from the mean + 2×SD assuming normal distribution

In analysing lateral thermal spread by tissue type, the 2 studies which looked at appendicectomy demonstrated very narrow widths of lateral thermal spread [33, 38], as did the study looking at peritoneal incision [42]. Whilst those looking at mesenteric vessels, gynaecological operations or pulmonary vessels quoted much higher results. The results between devices in all of these studies were similar suggesting that these differences may be due to study methodology rather than true differences in lateral thermal spread due to the tissue type being studied.

Notably as addressed by Mannelli and colleagues, utilising energy devices, heating of the target tissue and subsequent fixation and embedding often leads to shrinkage and retraction of the tissue [40]. Balachandran and colleagues also noted smaller vessel diameters following fixation then when measured prior to this step but did not account for this in their lateral thermal spread analysis [22]. This highlights concern that the true extent of lateral thermal spread by surgical energy devices may be underrepresented in the literature.

Considering the various approaches to measuring lateral thermal spread, it is suggested that a collagenous staining technique such as Masson’s Trichrome or EVG is utilised by future studies. This will allow simple protocols and cost-effective study of tissue injury. Agreed protocols will facilitate the directly comparable data required for future systematic analysis and avoid potentially confounding steam plumes as seen with thermography and the expense and expertise needed for DNA labelling methodologies.

## Conclusions

There are little data and significant heterogeneity in the testing methodology and outcome reporting for the assessment of the effects of surgical energy devices on human tissue. Most burst pressure studies in human tissues are congruent with animal studies, showing most devices form vessel seals with a bursting pressure above physiological levels; however, there is significant variation between tissues and devices with a proportion of seal failure or burst pressures below physiological blood pressure levels which could pose a clinical risk of haemorrhage with no clear correlation between tissues or device types.

Lateral thermal spread studies also show significant heterogeneity with a large range of values quoted between devices and tissue types. There are also large differences in values quoted using thermography or TUNEL staining compared with conventional H&E staining. Clear methodologies for the assessment of lateral thermal spread need to be developed to allow comparison between studies and devices and these should include consideration to confounding variables such as tissue retraction following fixation.

Due to heterogeneity between studies in both methodology and reporting, no device or tissue specific conclusions can be drawn and no statistical comparison is possible, highlighting the need for standardised methodologies.

## Supplementary Information

Below is the link to the electronic supplementary material.

Supplementary file1 (DOCX 116 KB)

## Data Availability

The authors confirm they are happy to supply all data produced during this review available to researchers upon request.

## Abbreviations

ABP: Advanced bipolar device
CASP: Critical appraisal skills program
EVG: Elastica van Gieson
H&E: Haematoxylin and Eosin
IMA: Inferior mesenteric artery
JBI: Joanna Briggs Institute
PRISMA: Preferred reporting items for systematic reviews and meta-analyses
SAGES: Society of American Gastrointestinal and Endoscopic Surgeons
USAD: Ultrasonically activated device
