# Supplementary material for: Tissue effects of advanced energy devices: a systematic review and descriptive analysis of vascular sealing and lateral thermal damage in human tissue
Source: Surg Endosc. 2026 Oct 13;40(11):9431–42. doi: 10.1007/s00464-026-13485-y (PMC13755015; doi:10.1007/s00464-026-13485-y)
Supplement: Supplementary file 1 [file 464_2026_13485_MOESM1_ESM.docx]

**Appendices**

**Appendix 1 – Search strategy for Ovid Medline and Embase**

Ovid MEDLINE(R) <1996 to  October Week 4 2025>

Embase <1996 to 2025 Week 44>

1	(advanced bipolar or abpd or EBVS or electrosurgical bipolar vessel seal* or ligasure* or enseal* or thunderbeat* or powerseal or vessel sealing or bipolar vessel sealing).mp. [mp=ti, bt, ab, ot, nm, hw, fx, kf, ox, px, rx, ui, sy, ux, mx, tn, dm, mf, dv, dq]	6953

2	(ultrasonically activated device or Ultrasonic coagul*or ultrasonic vessel sealer or ultrasonic activated device or USAD or ultrasonic device or ultrasonic sealing or harmonic* or sonicision* or ultracision*).mp. [mp=ti, bt, ab, ot, nm, hw, fx, kf, ox, px, rx, ui, sy, ux, mx, tn, dm, mf, dv, dq]	55394

3	1 or 2	61432

4	(lateral thermal spread or lateral thermal damage or lateral thermal injury or lateral spread or lateral damage or lateral injury or thermal damage or thermal injury or thermal spread or burst pressure or bursting pressure or vessel seal*).mp. [mp=ti, bt, ab, ot, nm, hw, fx, kf, ox, px, rx, ui, sy, ux, mx, tn, dm, mf, dv, dq]	26743

5	3 and 4	4209

6	limit 5 to English language	4112

7	limit 6 to humans	3226

8	remove duplicates from 7	2750

9	limit 8 to (adaptive clinical trial or case reports or classical article or clinical study or clinical trial, all or clinical trial or comparative study or controlled clinical trial or "corrected and republished article" or journal article or meta-analysis or multicenter study or network meta-analysis or observational study or pragmatic clinical trial or randomized controlled trial or "review" or "scoping review" or "systematic review" or article or article in press)	1967

**Appendix 2 – Search strategy for Scopus**

(TITLE-ABS-KEY ( "Bipolar sealing" OR ligasure* OR "vessel seal*") OR TITLE-ABS-KEY (ultrasonic shears OR ultrasonic OR harmonic OR USAD ) AND TITLE-ABS-KEY ("lateral thermal" OR "lateral damage" OR "burst pressure" OR "bursting pressure") )”

272 results

Appendix 3 – Risk of Bias assessments by study design

| **Randomized controlled trials (ROB2)** | | | | | | | |
| --- | --- | --- | --- | --- | --- | --- | --- |
| Author (Year) | D1 | D2 | D3 | D4 | D5 | Overall risk | |
| Gorginzadeh et al (2024)[16] | Low | Some concerns | Low | Low | Low | Some concerns | |
| Mihanovic et al (2021)[30] | Low | Low | Low | Low | Low | Low | |
| Choi et al (2018) [13] | Low | Low | Low | Low | Low | Low | |
| Caltekin et al (2017) [8] | Some concerns | Low | Low | Low | Low | Some concerns | |
| Pogorelic et al (2017)[36] | Low | Low | Low | Low | Low | Low | |
| Toishi et al (2014)[43] | High | High | Low | Low | Low | High | |
| Zhu et al (2012)[48] | Some concerns | Low | Low | Low | Low | Some concerns | |
| Druzijanic et al (2012)[14] | Some concerns | Low | Low | Low | Low | Some concerns | |
| Noble et al (2011)[34] | Low | Low | Low | Low | Low | Low | |
| *D1 – Randomization process, D2 Deviations from intended interventions, D3 – Missing outcome data, D4 – Measurement of the outcome, D5 – Selection of the reported result* | | | | | | | |
| **Non-randomised trials (ROBINS-I v2)** | | | | | | | |
| Authors (Year) | D1 | D2 | D3 | D4 | D5 | D6 | Overall risk |
| Turkan et al (2019)[45] | Low | Low | Low | Low | Low | Low | Low |
| Yoshiya et al (2018) [47] | Low | Low | Low | Low | Low | Low | Low |
| Balachandran et al (2017) [2] | Serious | Low | Low | Critical | Low | Low | Critical |
| Adamczewski z et al (2015)[1] | Low | Low | Low | Low | Low | Low | Low |
| Liberman et al (2015)[26] | Low | Low | Low | Low | Low | Low | Low |
| Liberman et al (2014)[25] | Low | Low | Low | Low | Low | Low | Low |
| Bibi et al (2014)[4] | Low | Low | Low | Low | Low | Low | Low |
| Brezezinski et al (2011)[7] | Serious | Low | Low | Low | Low | Low | Serious |
| Tsunezuka et al (2010)[44] | Low | Low | Low | Low | Low | Low | Low |
| Zorn et al (2010)[49] | Low | Low | Low | Low | Low | Low | Low |
| Ching et al (2008)[12] | Low | Low | Low | Low | Low | Low | Low |
| Kajiyama et al (2005)[46] | Low | Low | Low | Low | Low | Low | Low |
| *D1 – Confounding, D2 – Classification of intervention, D3 – Selection of participants, D4 – Missing data. D5 – Measurement of the outcome , D6 – selection of the reported result* | | | | | | | |
| **Case Series (JBI checklist)** | | | | | | | |
| Author (Year) | Details | | | | | | Overall risk |
| Kawamata et al (2001)[20] | Clear protocol of testing samples however no attempt to account for confounding or details of recruitment of patients | | | | | | Moderate |
| **Case-controls/retrospective analyses/cadaveric (CASP checklist)** | | | | | | | |
| Author (Year) | Details | | | | | | Overall risk |
| Latimer et al (2014)[23] | Clear design and protocol. Most confounders accounted for. Possible confounding from ex vivo component of study not accounted for | | | | | | Low |
| Mannelli et al (2014)[27] | Clear design and protocol. Clearly defined control and thermal injury definition. | | | | | | Low |
| Lesser et al (2013)[24] | Clear design and protocol. Confounders considered. Limited information on recruitment process. | | | | | | Low |
| Hanby et al (2011)[17] | No details given for methodology of lateral thermal spread analysis or details for cadaveric material. At significant risk of confounding | | | | | | High |
| Modi et al (2009)[32] | Clear protocol of testing however potential for confounding between surgeons not addressed, large discrepancy between number of specimens between groups | | | | | | Moderate |
| Miralles et al (2023)[31] | Samples are pair matched though no attempt to mitigate for selection bias. No discussion on effects of frozen specimens on outcomes. At risk of confounding | | | | | | Moderate |

Appendix 3 – Risk of bias assessments for all included studies. Listed by study design to allow grouping by assessment tool. Domains listed as per assessment tool protocols with overall assessment of risk in final column.

**Appendix 4 – Study characteristics for studies including bursting pressure analysis**

| **Authors (Year)** | **Tissue/speciality** | **In vivo/ ex vivo** | **Study design** | **Number of patients in study** | **Sample size for energy device arm** |
| --- | --- | --- | --- | --- | --- |
| **Kajiyama et al (2005)[46]** | Left gastric artery | ex vivo | single centre cohort | 27 | 27 |
| **Ching et al (2008)[12]** | Varicose veins | ex vivo | cohort | 15 | 156 |
| **Tsunezuka et al (2010)[44]** | Pulmonary vessels | in vivo | cohort | 84 | 25 |
| **Noble et al (2011)[34]** | Mesenteric vessels | ex vivo | single centre RCT | 18 | 93 |
| **Lesser et al (2013)[24]** | Pulmonary vessels | in vivo | Single centre RCT | 30 | 30 |
| **Latimer et al (2014)[23]** | Multiple sites | Cadaveric |  | 6 | 185 |
| **Liberman et al (2014[25])** | Pulmonary vessels | ex vivo | prospective cohort | 14 | 49 |
| **Toishi et al (2014)[43]** | Pulmonary vessels | in vivo | single centre RCT | 97 | 48 |
| **Bibi et al (2014)[4]** | IMA | In and ex vivo | cohort | 25 |  |
| **Liberman et al (2015)[26]** | Pulmonary vessels | ex vivo | prospective cohort, pre-clinical feasibility study | 43 | 90 |
| **Balachandran et al (2017)[2]** | IMA | in and ex vivo | single centre cohort | 30 | 162 |
| **Miralles et al (2023)[31]** | Varicose veins | in vitro | Case-control | 30 | 20 |

**Appendix 5** - Descriptive statistics of studies reporting any form of lateral thermal spread analysis when using surgical energy devices

| Authors (Year) | Tissue | in/ex vivo | Study design | No of patients | Sample size in energy device arm |
| --- | --- | --- | --- | --- | --- |
| Gorginzadeh et al (2024)[16] | Hysterectomy | in vivo | single centre, RCT | 39 | 156 |
| Mihanovic et al (2021)[30] | Appendix | in vivo | Two centre RCT | 49 | 98 |
| Turkan et al (2019)[45] | Breast | in vivo | Cohort | 30 | 30 |
| Yoshiya et al (2018)[47] | Lung | in vivo | prospective cohort | 20 | 43 |
| Choi et al (2018)[13] | Hysterectomy | in vivo | single centre RCT | 20 | 20 |
| Caltekin et al (2017)[8] | Hysterectomy | in vivo | single centre RCT | 18 | 72 |
| Balachandran et al (2017)[2] | IMA | in and ex vivo | single centre cohort | 10 | 30 |
| Pogorelic et al (2017)[36] | Appendix | in vivo | single centre RCT | 99 | 99 |
| Adamczewski et al (2015)[1] | Thyroid | in vivo | Cohort | 73 | 73 |
| Bibi et al (2014)[4] | IMA | In and ex vivo | Cohort | 25 | 54 |
| Mannelli et al (2014)[27] | Head and neck | Ex vivo | Cohort | 10 | 10 |
| Zhu et al (2012)[48] | Myometrium | ex vivo | Single centre RCT | 15 | 15 |
| Druzijanic et al (2012)[14] | Peritoneum | in vivo | single centre RCT | 100 | 100 |
| Hanby et al (2011)[17] | Tongue | ex vivo (cadaveric) |  | 2 | 3 |
| Noble et al (2011)[34] | Mesenteric vessels | ex vivo | single centre RCT | 18 | 93 |
| Brezezinski et al (2011)[7] | Thyroid | in vivo | Single centre cohort | 76 | 760 |
| Zorn et al (2010)[49] | Prostate | in vivo | cohort | 10 | 10 |
| Modi et al (2009)[32] | Adenoid tonsil | in vivo | retrospective analysis | 228 | 46 |
| Ching et al (2008)[12] | Varicose veins | ex vivo | cohort | 15 | 41 |
| Kawamata et al (2001)[20] | Brain | in vivo | Case-control | 8 | 8 |
